# Integrating nurse-led Self-Management Support (SMS) in routine primary care: design of a hybrid effectiveness-implementation study among type 2 diabetes patients with problems of daily functioning and emotional distress: a study protocol

**DOI:** 10.1186/1471-2296-14-77

**Published:** 2013-06-07

**Authors:** Anneke van Dijk - de Vries, Marloes A van Bokhoven, Berend Terluin, Trudy van der Weijden, Jacques Th M van Eijk

**Affiliations:** 1 Department of Family Medicine, CAPHRI School for Public Health and Primary care, Maastricht University, PO Box 616, Maastricht 6200 MD, The Netherlands; 2 Department of Social Medicine, CAPHRI School for Public Health and Primary care, Maastricht University, Maastricht, The Netherlands; 3 Department of General Practice, EMGO Institute for Health and Care Research, VU University Medical Center, Amsterdam, The Netherlands

**Keywords:** Hybrid Effectiveness–Implementation Design, Primary Care, Chronic Disease, Psychosocial Problems, Self-Management, Practice Nurse

## Abstract

**Background:**

Psychosocial problems are more prevalent among patients with chronic diseases than among the general population. They may lead to a downward spiral of poor adherence, deterioration of the condition and decline in daily functioning. In addition to medical management, systematic attention to emotional and role management tasks during routine chronic care seems mandatory. We intend to integrate an existing nurse-led minimal psychological intervention to support patients’ self-management, which appeared to be effective and cost-effective, in routine care by primary care nurses, so we adjusted it to fit the host setting. The resulting Self-Management Support (SMS) programme involves early detection of patients with emotional distress and problems of daily functioning, as well as self-management support through problem solving and reattribution techniques. Strategies to embed SMS in daily practice include training and booster sessions for practice nurses as well as organisational and financial arrangements. This study aims to simultaneously evaluate the implementation process and effects of SMS in routine care, using a hybrid effectiveness–implementation design.

**Methods/Design:**

Registration data, questionnaires and interviews will be used to explore the facilitators, barriers and costs regarding successful implementation of SMS. The effects of SMS will be evaluated in a pragmatic cluster-randomised controlled trial with a baseline measurement and follow-up measurements after 4 and 12 months. The population will consist of 46 practice nurses and their type 2 diabetes patients (N = 460; 10 per practice nurse). The practice nurses will be randomly assigned to the intervention or control group. Practice nurses of the intervention group will receive SMS training. Patients for the intervention and control groups will be recruited by a researcher-led self-administered screening procedure to decide which patients of those scheduled for routine consultation are likely to be detected by the practice nurses as eligible for the self-management support. Primary outcome measure is patients’ daily functioning. Secondary measures include emotional well-being, participation, autonomy and control over the disease.

**Discussion:**

Our hybrid study design is complicated by the detection method used by the practice nurses. This method is an implementation issue in itself that has consequences for the realisation and power of the effect evaluation.

**Trial registration:**

Current Controlled Trials, NTR2764

## Background

The prevalence rate of psychosocial problems is higher among patients with chronic somatic diseases than among the general population [1-4]. Problems like depression or distress may hamper the ability to manage a chronic condition [5]. A reciprocal relationship has been suggested: emotional distress may interfere with control over the disease, and at the same time, poor control over the disease can lead to emotional distress. Persistent negative feelings have implications for adherence to medication and lifestyle regiments, motivation, self-efficacy and problem solving [6,7]. Patients therefore face not only the day-to-day medical management, but also the challenge to deal with their emotions and problems of daily functioning in order to remain in control of their illness [6,8]. To improve the outcomes of chronic care, Lorig and Holman argue that three self-management tasks should be addressed in primary care: medical management, emotional management and role management [8]. However, Dutch guidelines for the care of chronic diseases like type 2 diabetes mellitus primarily focus on medical and lifestyle management [9,10]. This seems too narrow a focus, as there is also room for improvement in the detection and treatment of emotional problems in the chronically ill [4,11,12].

Our study intends to facilitate a shift towards a chronic care approach that combines attention to both biomedical and psychosocial aspects. We believe that equipping practice nurses (PNs) with the ability to provide this might be an effective way to realise such a biopsychosocial approach in chronic care [13]. PNs in the Dutch primary care setting see patients on a regular basis, as they have the task of providing routine check-ups [14]. Since diabetes patients were the first to be cared for by PNs, we aim to integrate an existing nurse-led minimal psychological intervention (MPI) in the diabetes follow-up care by PNs. The nurse-led MPI has proved to be effective in supporting the self-management skills of diabetes and COPD patients with minor to moderate depression [15-18].

The MPI can only be adopted by PNs if a detection method is added that enables PNs to distinguish between patients who are active self-managers, patients who would benefit from the psychosocial support, and patients who need more specialised psychosocial diagnostics or treatment. We call the resulting stepped approach to detection and treatment ‘Self-Management Support’ (SMS). In addition to adapting the MPI to become SMS, we need to arrange for a training course with booster sessions, and ensure the allocation of time and organisational support to enable the PNs to adopt SMS in their current practice. For a thorough understanding of the effectiveness of SMS, the facilitators and barriers to its implementation in routine care need to be taken into account. Therefore, we want to start a hybrid effectiveness–implementation trial to examine the effectiveness of SMS in terms of patient outcomes, and focus on implementation outcomes.

### Aim

The aim of our study is to evaluate the process and effectiveness of SMS implemented as an integral part of the care for patients with type 2 diabetes mellitus provided by PNs. We will simultaneously address the following research questions:

1. *What is the uptake of the SMS programme by the practice nurses, and what barriers hamper the implementation of SMS in routine primary care?* (Study of the implementation strategies for SMS.)

2. *What is the effectiveness of SMS in terms of daily functioning, emotional health status, social participation, self-management behaviour, and health care use by patients with type 2 diabetes?* (Study of the SMS clinical intervention.)

The effectiveness of SMS (question 2) will be considered while taking the barriers to the implementation process (question 1) into account.

## Methods/Design

In our study, we will use a hybrid effectiveness–implementation design to focus on implementation issues and the effectiveness of SMS at the same time. Curran and colleagues have presented a continuum of three types of such hybrid effectiveness–implementation (E-I) study designs [19], ranging from effectiveness research with minimal implementation strategies (type 1) to designs where effectiveness and implementation are equally balanced (type 2) and an implementation approach with minimal focus on effectiveness outcomes (type 3). We will examine the effects of SMS on patient outcomes and simultaneously focus on implementation outcomes, hence it is a type 2 hybrid E-I study design.

Below, we first describe our implementation strategies (intervention) and then explain our evaluation methods regarding the implementation and effects of SMS in routine care (evaluation).

### Intervention

In a previous study, the nurse-led minimal psychological intervention (MPI) was carried out by trained research nurses [20]. In our present study, practice nurses who provide diabetes care will have to adopt the MPI in their current practice. This required changes with regard to the clinical intervention as well as the organisational context in which the intervention will be integrated [21]. This section explains the steps that will be taken to implement the resulting SMS in routine diabetes care.

#### Implementation strategy 1: adaptation of clinical intervention

The nurse-led MPI has been altered from a ‘research object’ to an intervention that can be systematically embedded in routine primary care. Adaptations were needed regarding the target group, providers, detection of eligible patients and self-management support. The shift from MPI to SMS is summarised in Table 1.

**Table 1:** The SMS approach contrasted with the original MPI

	**MPI**	**SMS**
Target group	Chronic patients with mild or non-severe major depression	Chronic patients with emotional distress and problems of daily functioning
Providers	Research nurses not involved in usual care	Practice nurses providing usual chronic care
Diagnostic approach	For research purposes. Using an extensive diagnostic interview, following strict psychiatric criteria	As part of daily care. Using simple tools, following a stepped care approach that fits the primary care setting
Self-management support intervention	1. cognitive therapy (reattribution)	1. problem solving
	2. problem solving	2. cognitive therapy (reattribution)

##### Target group

The original effective nurse-led MPI was provided specifically to patients with a diagnosis of depression, whereas a wide range of psychological and social problems occur in a routine primary care setting. What primarily matters in daily practice is to what extent patients experience problems of daily functioning [12]. SMS was therefore designed for chronic patients who encounter problems of daily functioning and emotional distress.

##### Providers

Instead of research nurses providing the intervention as an add-on to usual care, SMS will be integrated in usual care provided by PNs. PNs work in general practice under supervision of a GP. They see type 2 diabetes patients every 3 months for a diabetes check-up. SMS is to become an integral part of these check-ups.

##### Detection procedure

The diagnostic procedure that was used to recruit patients for the study to evaluate the original MPI is not feasible in routine primary care: it was limited to depression, and included an extensive and time-consuming diagnostic interview (the Mini International Neuropsychiatry Interview) to confirm a diagnosis of depression according to psychiatric criteria. For SMS, PNs will have to decide during routine diabetes check-ups whether watchful waiting, offering psychosocial self-management support or a referral to the GP because of symptoms of mental health problems is most appropriate. Therefore, we have introduced a stepped approach that allows interventions not sooner or more intense than necessary and not later or less intense than required. The approach is as follows. During each regular diabetes check-up, PNs will explore whether the patient is experiencing problems in daily life. Then they verbally administer the ‘Daily Functioning Thermometer’ (DFT), a visual analogue scale to rate the overall burden of diabetes. (See the Measurements section below for more information about the instruments.) Practice nurses will also verbally administer the 3-item Distress Screener (DS), a quick-scan instrument for emotional distress and an indicator of potential underlying severe mental health problems [22]. Patients with DS > 3 will be asked to complete the Four-Dimensional Symptom Questionnaire (4DSQ) [23] immediately after the consultation. This self-report measure is widely used in Dutch primary care to distinguish non-specific general distress from depression, anxiety and somatisation. Scoring the 50 items of the 4DSQ should take 5–10 minutes The completed 4DSQ will be returned to the PN, who will score the 4DSQ using a scoring form. Patient’s scores on the 4DSQ will distinguish between mild, moderate and severe mental health problems. Patients who experience problems of daily functioning (DFT > 4) and emotional health problems (DS > 3 combined with moderate scores on subscales of the 4DSQ) will receive self-management support from the PN. If a patient has only ‘mild’ scores on the 4DSQ, the PNs will opt for watchful waiting. The same option will be used for patients with moderate scores on the 4DSQ who do not report problems of daily functioning due to diabetes (DFT ≤ 4). These patients will be informed by telephone that there is as yet no indication for extra consultations. Patients with a ‘severe’ score on at least one of the subscales will be referred to the GP. The detection protocol is summarised in Table 2.

**Table 2:** Detection protocol

	**DFT** ≤ **4**	**DFT > 4**
DS ≤ 3	No indication	Watchful waiting
DS > 3	On the basis of the 4DSQ:	On the basis of the 4DSQ:
	Mild: watchful waiting	Mild: watchful waiting
	Moderate: watchful waiting	**Moderate: self-management support by PN**
	Severe: referral to GP	Severe: referral to GP

##### Self-management support programme

The self-management support by the PN will be provided during extra 20-minutes consultations. Its aim is to teach patients to take responsibility for the day-to-day management of their diabetes and its consequences. This self-management support is based on the MPI. The intervention strategy has been derived from principles of learning theory and has been described elsewhere [24]. PNs will help patients to define problems and solutions themselves, applying the techniques of problem-solving and reattribution. Problem-solving consists of 7 stages that efficiently address psychosocial problems and their possible solutions [25]. It starts by creating a link between emotional symptoms and problems, and explaining the rationale of the treatment: resolution of the problems may lead to resolution of the symptoms (step 1).

Step 2 involves clarifying and defining the problem. The third step is to set achievable goals. Once an achievable goal has been set, the patient will be asked to brainstorm about all potential solutions (step 4). Step 5 involves considering the pros and cons and selecting a preferred solution. In step 6, the solution will be implemented in daily life, and step 7 involves evaluating the progress. The problem-solving approach can easily be understood by patients and can also easily be taught to practice nurses [25]. Patients with strong emotional involvement will be supported using the reattribution technique. This starts by exploring and defining the patient’s problem, after which the patient is challenged to link cognitions to behaviour. To this end, patients may keep a diary to record thoughts, behaviours and related feelings. After possibilities to alter specific behaviours have been explored, an action plan will be formulated indicating how the goals can be achieved [20]. If specific problems appear to be persistent and serious over time, action will be undertaken to provide more specialised care. PNs are instructed to refer these patients to the GP.

In the original MPI, the cognitive approach appeared to benefit more highly educated patients more than lower educated ones [18]. The SMS will be implemented in a region in the south of the Netherlands that is characterised by relatively low socio-economic status [26], so in order to increase the benefit for patients with low socio-economic status, we will put greater emphasis on the problem-solving technique during the training of PNs.

**Figure 1:**
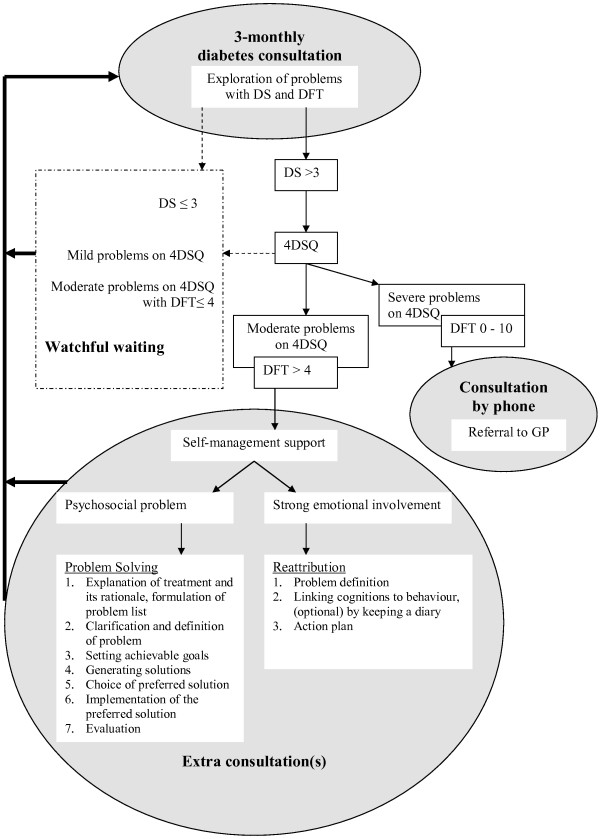
Flowchart for SMS intervention.

A flowchart of SMS is presented in Figure 1.

#### Implementation strategy 2: involvement of regional GP organisation

SMS will be implemented in one region in the south of the Netherlands. A group of 77 general practices (103 GPs) in the region, who collaborate in a GP organisation, was involved from the beginning of the SMS project, and they have prioritised the integration of SMS in the care for type 2 diabetes patients. The GPs are responsible for about 16,000 patients with type 2 diabetes mellitus. The region is characterised by relatively low socio-economic status, health status and life expectancy and substantial ageing of the population [26,27]. Based on earlier research findings we estimate that as many as 2400 patients with type 2 diabetes mellitus (15%) may suffer from emotional distress accompanied by problems of daily functioning [28].

#### Implementation strategy 3: financial support

The reimbursement of diabetes care in the Netherlands is regulated by means of diagnosis-treatment combinations (DTC), a Dutch variant of the Diagnosis Related Groups [29]. For each care group, health insurers’ purchase integrated care by negotiating a fixed price per patient per year. The elements of care with corresponding tariffs are defined in a shared care protocol. This is driven by the Dutch ‘care standard’ for diabetes [9], which is based on existing guidelines, protocols and performance indicators. During the study, a health insurance company will reimburse the extra time spent on SMS by practice nurses and GPs. The reimbursement will be integrated in the DTC for diabetes care.

#### Implementation strategy 4: training

The PNs in the intervention group will be trained to carry out SMS in three 8-hour training sessions. The training programme is based on a nurses’ training course that has been shown to be feasible, attractive and successful. PNs learn how to help patients identify their problems and set achievable goals [24]. The training programme will also focus on exploration skills. PNs will learn to use instruments to establish the severity of psychosocial problems in a stepped approach (first DFT and DS, and if appropriate further exploration using 4DSQ). The training sessions will take place in small groups, to facilitate active participation of the PNs, for example by role-play. These three training sessions will be followed up by booster sessions and telephone consultations, whose frequency will depend on the PNs’ needs. Booster sessions will be used to maintain and improve the PNs’ SMS skills. PNs will make audiotapes during consultations, which will be reflected on during booster sessions.

#### Implementation strategy 5: registration system for SMS

SMS will be integrated in the registration system that GPs and PNs use for diabetes care. The steps to be taken during the detection phase of SMS as well as the different stages of the self-management support by the PN are displayed in the regular registration system. PNs will record how patients rate their burden of diabetes (DFT), as well as their scores on the distress screener (DS). This information will be displayed at the next diabetes check-up. The system also helps PNs compute the outcomes of the 50-item Four-Dimensional Symptom Questionnaire (4DSQ). The PNs will also record the self-management support steps that are taken during each consultation, and the patients’ action plans.

### Evaluation of the implementation process

#### Design

A process evaluation will be done to analyse the facilitators and barriers regarding integration of SMS in routine care. We will gather quantitative and qualitative data about the intervention as an integral part of the routine diabetes care, as well as about the health professionals (PNs and GPs) and patients, and the impact of SMS on the practice organisation and the community [30]. How SMS has been implemented and received by the participants will be measured by means of the following concepts, as recommended by Saunders and colleagues [31]: fidelity, dose delivered, dose received, reach, recruitment and context. In addition, the costs of implementing SMS in routine care will be assessed. The process evaluation will include formative evaluation with respect to the new components of the clinical intervention, as these aspects may need to be refined or optimised to make them fit the local setting.

#### Variables and measurements

* Fidelity* refers to the extent to which SMS has been implemented as planned. At each diabetes consultation, the PNs will record which steps of the SMS protocol have been carried out. The outcomes on the DS, DFT and 4DSQ, the choices that have been made regarding further treatment and the patients’ action plans will also be recorded. The registration system also offers room for comments. PNs will be asked to give an explanation if they deviate from the SMS protocol.

*Dose delivered* is measured as the number of components of SMS per patient that have been carried out as intended. Data will be derived from the registration system.

*Dose received* includes two components: *satisfaction* and *exposure.*

Exposure refers to the extent to which participants (patients and health professionals) are actively engaged with the SMS programme and use the tools of SMS in daily practice. Engagement and satisfaction of participants will be measured among the providers (PNs and GPs) and among the patients.

PNs will be asked to complete an evaluation form after the training sessions, with questions about the clarity of wording of the components of the training course. With regard to the integration of SMS in routine diabetes consultations, issues discussed with PNs during booster sessions will be recorded during the follow-up period. This will provide qualitative information about the barriers and facilitators that PNs may experience. At the end of the follow-up period (one year after the SMS training course), we will develop a questionnaire including all the barriers and facilitators that have been discussed during the booster sessions. PNs will be asked to individually rate all these issues.

The engagement and satisfaction of GPs will be evaluated by means of self-administered questionnaires. All participating GPs from the intervention arm will be asked about their involvement in the SMS programme and their views on the impact of SMS for their practice.

How patients experience being involved in the SMS programme will be explored in detail by holding semi-structured interviews with a purposive sample of patients who have received the SMS from their PN. These interviews will be held during the follow-up period. The topics include patients’ experiences with the detection phase of SMS, the self-management support, the opportunities for and barriers to the implementation of their action plans in daily life, and their suggestions for improvement of the SMS. Interviews with patients will be audio-taped and transcribed verbatim.

*Recruitment* refers to the way we have approached and invited the PNs and GPs to become involved in the project to implement and evaluate SMS.

*Reach* refers to the participation rate and will be measured as the number of GPs and PNs who actually participated.

*Context* refers to the factors relating to the practice, the community, the social/political context, or other situational issues that affect either the implementation of SMS or its outcomes. To examine context issues in terms of the implementation and effect of SMS, we will draw up the minutes of meetings with the regional care group of general practitioners, the health insurer and our research team.

#### Cost evaluation

We will measure the direct costs of SMS. In addition to the training and booster sessions, these will include the time to integrate SMS in routine diabetes consultations. PNs will record the time they spend on each stage of the stepped SMS approach.

#### Data analysis

Data of the implementation study will be analysed by means of descriptive statistics.

The qualitative interviews with patients will be analysed by interpretative phenomenological analysis, a framework that can be used to develop in-depth descriptions of patients’ experiences. The purpose is to explore the patients’ perceptions of what is important in relation to SMS. We will abstract themes and cluster them for each case [32]. Abstracting and clustering of the themes will be done independently by two researchers.

### Clinical effect evaluation

#### Design

The effect evaluation of SMS involves a pragmatic 2-armed cluster-randomised controlled trial (cluster-RCT) with PNs as the unit of randomisation. Allocation concealment will be achieved by having an independent experienced research assistant perform a blockwise randomisation using a random number seed computer program. PNs (n = 46) will be assigned to an intervention group or control group at an allocation ratio of 1:1, after stratification into PNs working alone in a practice, PNs working in a team and PNs working in different practices. PNs working in the same general practice will be randomised to the same trial arm.

PNs in the intervention arm will be trained to apply SMS in addition to the usual care, which consists of 3-monthly diabetes check-ups according to the current guidelines [10]. PNs in the control arm will be instructed to provide usual care.

Patients with emotional distress and problems of daily functioning will be included in the effect evaluation. To maintain the balance between the intervention and control groups, a screening procedure will be carried out by the research centre. A self-administered postal questionnaire will be used to detect eligible patients.

Approval for this study has been obtained from the Medical Ethics Committee of Maastricht University/University Hospital Maastricht.

#### Sample size calculation

Assuming an alpha of 0.05 and a beta of 0.90, an improvement in perceived daily functioning (defined as a score less than or equal to 4 on the Daily Functioning Thermometer (DFT), our primary outcome) at T12 occurring in 20% of the patients in the intervention group versus 5% of those in the control group requires at least a net number of 116 patients per arm (N = 232; 5 patients per practice nurse). It will be necessary to take account of a possible dependence between observations on patients of the same practice nurse. The intra-class correlation coefficient (ICC) is assumed to be 0.04, a median value for cluster-RCTs in the primary care setting [33]. Assuming a 30% loss to follow-up [17] we need to recruit at least 331 patients (8 per PN). Since participation in the screening procedure will not necessarily mean that patients also give informed consent for the effect evaluation, 10 consecutive patients for each PN will be invited to participate in the effect evaluation (N = 460).

#### Patient recruitment

All patients with a clinically established diagnosis of type 2 diabetes mellitus who are able to read and write Dutch will be sent a letter by their GP, explaining SMS and asking for informed consent to participate. Patients will receive this letter six weeks before a planned diabetes consultation with their PN. The enclosed screening questionnaire will contain the same instruments that PNs in the intervention group are going to use to detect whether patients are eligible for the self-management support, i.e. the ‘Daily Functioning Thermometer’ (DFT) and the 3-item Distress Screener (DS) [22]. For more information about these instruments, see the section on Measurements below. Patients will be asked to return the completed questionnaire to the research centre and give informed consent for their data to be used for recruitment if they are found to be at risk. For each practice nurse, 10 consecutive patients with scores of DFT >4 and DS >3 will be asked to participate in the study and to give informed consent for the follow-up measurements. To prevent bias, PNs will not be notified about the outcomes of the recruitment procedure.

**Figure 2:**
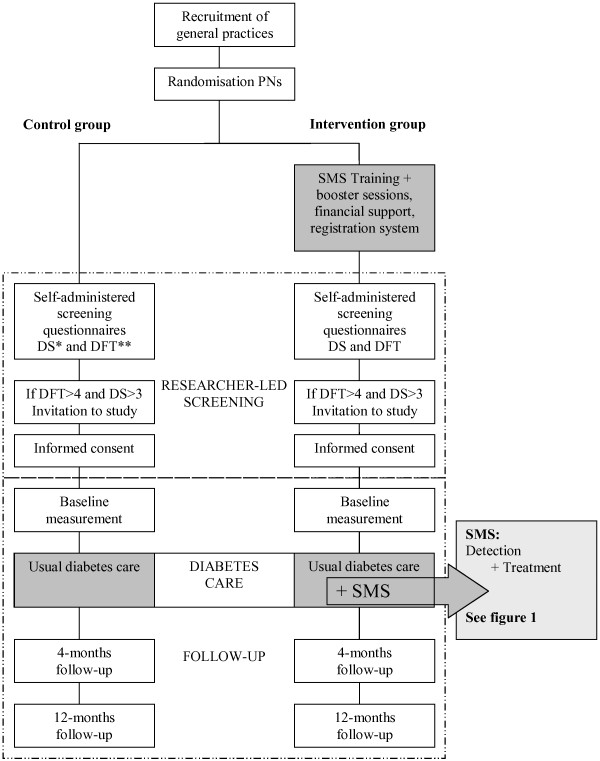
**Flowchart for SMS trial.** * DFT = Daily Functioning Thermometer. ** DS = Distress Screener.

A flowchart of the trial is shown in Figure 2.

#### Measurements

To assess the effectiveness of SMS, follow-up measurements will be carried out at baseline and 4 and 12 months after inclusion. Table 3 provides an overview of the measures of the effect evaluation and the times of assessment.

**Table 3:** Effect evaluation: outcome measures and time of assessment

**Concept**	**Measurement**	**Items**	**Moment in time**			
			**3-monthly diabetes consultations during follow-up**	**T0**	**T-4**	**T-12**
*Primary outcome*						
Daily functioning	DFT	1		*	*	*
*Secondary outcomes*						
Diabetes-related emotional distress	PAID	20		*	*	*
Mental health problems	PSYCHLOPS	9	*^2^	*^1^	*	*
	4DSQ	50			*	*
Quality of life	SF-12	12		*	*	*
Participation and autonomy	IPA	32 + 9		*	*	*
Job performance	SF-HLQ	5		*	*	*
Self-management knowledge and behaviour	PIH-NL	12		*	*	*
General self-efficacy	GSES-12	12		*	*	*
Control over the disease	Average blood glucose level (HbA1c)	1	*			
Health care use		5		*	*	*
*Covariates*						
Patient demographics	Sex	1		*		
	Age	1		*		
	Year of diagnosis	1		*		
	Treatment of diabetes	1		*		
	Marital status	1		*		
	Education	1		*		
Practice nurse demographics	Age	1		*		
	Training	1		*		
	Type of general practice(s) (one GP, two GPs, group, health centre)	1		*		

1 Only patients in intervention arm. Risk of contamination.

2 Only eligible patients (Distress Screener > 3).

* = measuring moment.

##### Background variables

Patient variables that will be reported are sex, age, year of diagnosis, treatment of diabetes (insulin therapy or tablets), marital status and education. Furthermore, we will record the age of the PNs, the number of PNs for diabetes management working in a general practice, as well as the educational background of the PNs.

##### Primary outcome measure

The primary outcome will be daily functioning as measured by means of the Daily Functioning Thermometer (DFT). This is a visual analogue scale (VAS) on which patients can rate the overall burden of diabetes on their daily functioning by indicating a position along a continuous vertical 100 mm line between 0 (‘no burden at all’) and 10 (‘extreme burden’). The VAS score is determined by measuring the distance to the point marked by the patient, in millimetres. The DFT is comparable to the Distress Thermometer (DT), an easily understood, valid and feasible self-report measure of distress used among various groups of cancer patients [34]. A cut-off score of 4 has been chosen to differentiate between patients who could benefit from self-management support by the PN (DFT > 4) and patients who have apparently found a satisfactory way to live with the consequences of their diabetes (DFT ≤ 4). A pilot study among 7 patients with type 2 diabetes mellitus confirmed the face validity of the DFT.

##### Secondary outcome measures

Three instruments will assess patients’ emotional health status.

Diabetes-related emotional distress will be measured by means of the Problem Areas in Diabetes questionnaire (PAID). This 20-item scale (with five-point Likert scales) describes common problematic situations for diabetes patients, each representing a unique area of diabetes-specific emotional distress. The sum of the scores for the 20 items is multiplied by 1.25 to get a final score between 0 and 100. The Dutch PAID has good internal consistency and validity [35-37].

The Four-Dimensional Symptom Questionnaire (4DSQ) will be used to measure the presence and severity of mental health problems [23]. The levels of distress (16 items), depression (6 items), anxiety (12 items) and somatisation (16 items) will be assessed. The reference period is ‘the past week’. The item scores are summated to obtain scale scores. The 4DSQ has been found to be a valid self-reported questionnaire in the primary care setting [23]. For a more qualitative evaluation of emotional problems that patients experience, we will use the Psychological Outcome Profiles (PSYCHLOPS). The PSYCHLOPS asks patients to describe and score their own emotional health problems. It is a validated patient-generated measure with pre-therapy, during-therapy and post-therapy versions. The instrument is intended to measure individual changes. It is responsive to change and is internally consistent [38].

Both PSYCHLOPS and 4DSQ explicitly expect patients to define and rate their emotional health problems. An increased awareness about their emotional problems of daily functioning could influence the issues discussed during the diabetes consultation, which could result in reduced contrast between the two groups. We will not use the 4DSQ and the PSYCHLOPS in the control group at T0, as the timing of the baseline measurement is just before a diabetes consultation, whereas the assessment at T4 is further removed from a diabetes consultation. Inclusion of the PSYCHLOPS and 4DSQ at both T4 and at the end of the follow-up period (T12) will provide insight into individual changes. In the intervention group, inclusion of the PSYCHLOPS at baseline will provide valuable information about the patients’ individual emotional problems. The 4DSQ does not need to be included in the baseline measurement as it is already part of the SMS intervention.

Quality of life, i.e. functional health and well-being from the patient’s point of view, will be measured by means of the 12-item Short-Form Health Survey (SF-12), a validated subset of the generic health status questionnaire SF-36 [39,40]. Data will be summarised in 2 scales: a physical component summary (PCS) and a mental component summary (MCS).

Participation and autonomy will be measured by means of the Impact on Participation and Autonomy (IPA) questionnaire. The IPA has proved to be valid, reliable and responsive to change [41]. Thirty-two items (five-point Likert scales) cover five domains of participation: ‘autonomy indoors’, ‘family role’, ‘autonomy outdoors’, ‘social life and relationships’ and ‘work and education’. A score for each subscale will be calculated. In addition, 9 items will evaluate the extent to which limitations are experienced as problematic: ‘no problems’, ‘minor problems’ or ‘major problems’. These items will be scored separately [42].

Five items from the Short-Form Health and Labour Questionnaire (SF-HLQ) will be used to measure difficulties of job performance due to health problems. The SF-HLQ is a generic and validated measurement to collect data about productivity loss relating to health problems [43].

Patients’ self-management knowledge and behaviours regarding their diabetes will be measured by the Partners in Health (PIH) scale [44]. This scale reliably and validly measures aspects of patient progress within a self-management programme for a chronic condition. We will use the Dutch version (PIH-NL). The 12 items (0–8 Likert scales with 0 indicating high self-management and 8 low self-management) cover four domains of patients’ competency in relation to the self-management of their chronic condition (knowledge, coping, management of condition and adherence to treatment).

The General Self-Efficacy Scale (GSES-12) will be used to assess the patients’ belief in their ability to organise and engage in certain behaviours. The sum score of the 12 items (five-point Likert scales) reflects an internally consistent and stable unidimensional construct of general self-efficacy [45,46].

At each diabetes consultation, the blood glucose level over the past 2 to 3 months (HbA1c) will be measured and recorded in the registration system.

The effect of SMS on patients’ health care utilisation refers to individual numbers of visits to the general practice, specialist referrals and admissions, mental health care consultations, use of home care and hours of paid and unpaid household help. Patients will record their health care use retrospectively (over the last month) in the patient questionnaires administered at baseline and after 4 and 12 months. Data about referrals to the GP, and the number of contacts with the general practice will be collected from the GP’s registration system.

#### Data analysis of the effect evaluation

The effectiveness of SMS will be assessed by intention-to-treat analyses. The comparability of patients in the intervention and control groups regarding baseline characteristics and demographics will be described and tested for significant differences (t-tests and Chi-square tests). The average changes in DFT score will be calculated, as well as the number of patients that have DFT ≤4 after receiving SMS (T4 and T12). Separate scores will be calculated for the subscales of the secondary outcome measures. Changes in the primary and secondary outcomes between the intervention and control groups will be analysed using multilevel analyses (linear and logistic regression methods) as this will account for intracluster correlation among PNs. Multivariate regression analyses will be used to adjust the results for co-variables such as patient characteristics (age, sex, socio-economic status) and PN characteristics (age, educational background).

## Discussion

This paper describes a study protocol to implement and evaluate an evidence-based, nurse-led minimal psychological intervention (MPI) in routine diabetes care in the Dutch primary care setting. The intervention itself has already been found to be an effective and cost-effective method to improve self-management by chronic patients with mild to moderate depression, but its effect was evaluated in a trial with standardised conditions regarding the inclusion of patients, and the care was delivered by research nurses [20]. Both GPs and a health insurer have prioritised the inclusion of the MPI in routine diabetes care, so there was an ‘implementation momentum’ within the system [19]. Actually making this happen required adaptations to be introduced regarding providers, target group and detection procedure. The new approach has been named Self-Management Support (SMS).

### Our hybrid design and its potential

The effects of integrating SMS in the routine primary care setting can only be evaluated when it is implemented in the ‘real world’; the implementation strategies will be decisive for the success of SMS at the practice and patient levels. The essential adjustments to the intervention with regard to both providers and recipients require further insights into the effectiveness of SMS. An understanding of both the prerequisites of integrating SMS in routine care and the effectiveness of SMS is essential for future planning. In terms of the continuum of design types proposed by Curran and colleagues [19], our study is an example of a type 2 hybrid effectiveness–implementation (E-I) study design. The effects and the implementation process will be evaluated simultaneously.

Curran and colleagues [19] argue that the combination of two study designs in a hybrid study may have the potential to accelerate the translation of research into routine practice. They refer to the dominant approach of translating research into practice that starts with clinical efficacy research, then clinical effectiveness research, and finally implementation research. The potential for rapid integration of research into practice is also apparent in our study. SMS evolved from a joint endeavour of the regional care organisation of general practitioners, the health insurer and the evaluators. The intervention has been developed as a generic approach to support patients’ self-management. The outcomes of the implementation and evaluation of SMS will provide a broad perspective on barriers, facilitators and effects of the detection and treatment of psychosocial problems by PNs in chronic care. Hence, if SMS appears to be successfully implemented and has relevant effects, it could in the near future also be applied to patients with other chronic conditions. The shared vision of stakeholders regarding SMS and their involvement in the development and evaluation is expected to facilitate a rapid large-scale integration of SMS in routine care.

### Our hybrid design and its methodological challenges

In hybrid E-I studies, the world of implementation science has to combine with the tradition and vocabulary of researchers from clinical research backgrounds. This implies a complex balance in the design itself between internal validity and factors associated with implementation, but also can be an obstacle to funding and publication [19,47].

Our study involves an additional methodological issue with respect to hybrid design, viz. the new detection method that is introduced in our study to integrate the original MPI in routine care. Its feasibility in routine diabetes consultations is not yet known, and needs to be evaluated as part of our process evaluation. However, an adequate detection method is also a prerequisite for clinical effectiveness of SMS. The methodological issues regarding the detection method are further explained below.

In order to integrate the MPI in the daily care by practice nurses, we had to introduce a new detection method to fit the primary care setting. The MPI has been adapted to become a self-management intervention for patients with problems of daily functioning and emotional distress. As there was no primary care instrument available to detect patients with these psychosocial problems, we have introduced new instruments in the form of the Distress Screener and the Daily Functioning Thermometer, and defined the cut-off criteria. The Distress Screener and the Daily Functioning Thermometer are assumed to be valid for the early detection of patients with emotional distress and problems of daily functioning [22,34]. These instruments will be integrated into the routine diabetes consultations provided by PNs. Our implementation strategies (i.e. providing training, financial support and a registration system) will determine whether PNs are able to integrate the instruments in their daily practice. Evaluation of the implementation process will show what numbers of patients are detected, whether patients with problems of daily functioning (according to the DFT) and with moderate psychological problems (according to the Four-Dimensional Symptom Questionnaire, 4DSQ) are representative of the group of patients that are in need of the self-management support, and to what extent PNs have the skills to integrate the tools in their routine consultations. The feasibility, power and success of our detection method are unknown and will determine the success and feasibility of the whole SMS approach.

The detection method will an integral part of the tasks of the PNs during a routine diabetes consultation. PNs will be given a reasonable degree of freedom in applying the detection method in their consultations, as we use a pragmatic approach without strict internal controls. We aim to avoid interference of research activities with daily practice as much as possible, which has implications for the recruitment of patients for the effect evaluation.

In theory, all diabetes patients of the PNs would be eligible for participation in the effect evaluation of SMS, as the PNs will apply the detection part of SMS in every diabetes consultation. However, we have chosen to limit the effect evaluation to patients with actual problems of daily functioning and emotional distress. This will increase the contrast between the intervention and control groups, so we need to include patients that are likely to be detected during regular care and receive further treatment. We had to set up a researcher-led parallel screening procedure to detect eligible patients from both the intervention and control groups. Based on our pragmatic approach, the conditions for this screening procedure are:

1. The self-administered screening questionnaire is sent to the patient’s home address and includes the instruments that PNs apply during consultation.

2. The screening does not interfere with daily practice in the control arm. As the DFT and DS consist of simple and generic questions, we do not expect any interference with daily care.

3. The time interval between the researcher-led screening and the nurse-led detection procedure needs to be limited. We have decided to send the screening questionnaire six weeks before the individual diabetes consultation appointment.

However, we cannot avoid the risk that natural fluctuations in the emotional health status of patients will result in different outcomes on the DFT and DS in the nurse-led detection compared to the researcher-led postal screening. The time interval could become too long, for example if a consultation appointment is cancelled. We thus have to take into account that conclusions based on the researcher-led screening questionnaires will not always correspond to those of the same instruments applied by the PNs during the consultations. As a consequence, a certain number of patients in our study sample will only be exposed to the detection part of SMS, without receiving follow-up treatment, while some patients who are not included in the effect evaluation may receive the self-management support. This may reduce the contrast between the intervention and control arms.

In conclusion, the strength of the clinical effectiveness evaluation in our study greatly depends on two risk factors of our hybrid design:

1. the extent to which PNs are able to detect patients who need the self-management support; and

2. the match between the patients who are detected by the researcher-led screening and those detected by the PN.

If PNs in our study would, for any reason, not detect or not treat patients who are recruited for the effect evaluation on the basis of the researcher-led screening, it may become difficult to demonstrate the clinical effectiveness of SMS. Nevertheless, the clinical effectiveness outcomes of SMS integrated in routine care are very important for decisions about future planning and (financial) support for SMS. If we are unable to demonstrate clinical effectiveness of SMS, the speed of translation of the evidence-based intervention into routine care may even be threatened due to the methodological issues of a hybrid effectiveness–implementation design. We will try to minimise the risks by providing training to PNs, followed by booster sessions, using a formative evaluation method that gives us the opportunity to refine the detection procedure, and by a researcher-led screening that closely resembles the detection at the consultation in terms of timing and procedure.

## Abbreviations

DFT: Daily functioning thermometer; DS: Distress screener; 4DSQ: Four-Dimensional Symptom Questionnaire; GP: General practitioner; Hybrid E-I design: Hybrid effectiveness–implementation design; MPI: Minimal psychological intervention; PN: Practice nurse; SMS: The Self-Management Support programme (detection and treatment).

## Competing interests

JvE is the initiator of the minimal psychological intervention (MPI). The 4DSQ was developed by BT. There are no financial competing interests.

## Authors’ contributions

JvE initiated this implementation project. All authors were involved in developing and refining the study design. AvD drafted the manuscript and prepared the revised versions. All other authors critically reviewed the draft revisions and read and approved the final manuscript.

## Pre-publication history

The pre-publication history for this paper can be accessed here:

http://www.biomedcentral.com/1471-2296/14/77/prepub

## References

[B1] Härter M Baumeister H Reuter K Jacobi F Höfler M Bengel J Wittchen H-U Increased 12-month prevalence rates of mental disorders in patients with chronic somatic diseases Psychother Psychosom 2007 76 354 360 10.1159/000107563 17917471

[B2] Ali S Stone MA Peters JL Davies MJ Khunti K The prevalence of co-morbid depression in adults with type 2 diabetes: a systematic review and meta-analysis Diabet Med 2006 23 11 1165 1173 10.1111/j.1464-5491.2006.01943.x 17054590

[B3] Van den Bemt L Schermer T Bor H Smink R Van Weel-Baumgarten E Lucassen P Van Weel C The risk for depression comorbidity in patients with COPD Chest 2009 135 1 108 114 10.1378/chest.08-0965 18689578

[B4] Pouwer F Should we screen for emotional distress in type 2 diabetes mellitus? Nat Rev Endocrinol 2009 5 12 665 671 10.1038/nrendo.2009.214 19884900

[B5] Jerant A Kravitz R Moore-Hill M Franks P Depressive symptoms moderated the effect of chronic illness self-management training on self-efficacy Med Care 2008 46 5 523 531 10.1097/MLR.0b013e31815f53a4 18438201

[B6] Fisher L Mullan JT Arean P Glasgow RE Hessler D Masharani U Diabetes distress but not clinical depression or depressive symptoms is associated with glycemic control in both cross-sectional and longitudinal analyses Diabet Care 2010 33 1 23 28 10.2337/dc09-1238 PMC2797978 19837786

[B7] DiMatteo MR Lepper HS Croghan TW Depression is a risk factor for noncompliance with medical treatment: meta-analysis of the effects of anxiety and depression on patient adherence Arch Int Med 2000 160 14 2101 2107 10.1001/archinte.160.14.2101 10904452

[B8] Lorig KR Holman H Self-management education: history, definition, outcomes, and mechanisms Ann Behav Med 2003 26 1 1 7 12867348 10.1207/S15324796ABM2601_01

[B9] Netherlands Diabetes Federation NDF care standard. Transparancy and quality of diabetes care for people with type 2 diabetes 2007 Amersfoort: Nederlandse Diabetes Federatie (NDF)

[B10] Rutten GEHM De Grauw WJC Nijpels G Goudswaard AN Uitewaal PJM Van der Does FEE Heine RJ Van Ballegooie E Verduijn MM Bouma M NHG-standaard diabetes mellitus type 2 (tweede herziening) [NHG practice guideline diabetes mellitus type 2 (second revision)] Huisarts Wet 2006 49 3 137 152

[B11] Van Eijk JTM Bosma H Jonkers CC Lamers F Muijrers EM Prescribing antidepressants and benzodiazepines in the Netherlands: is chronic physical illness involved? Depress Res Treat 2010 2010 Article ID 105931 6 10.1155/2010/105931 PMC2989733 21152218

[B12] Van Weel-Baumgarten E Lucassen P Hassink-Franke L Schers H A different way of looking at depression Int J Clin Pract 2010 64 11 1493 1495 10.1111/j.1742-1241.2010.02405.x 20846197

[B13] Van Dijk-de VA Moser A Mertens VC van der Linden J van der Weijden T van Eijk JT The ideal of biopsychosocial chronic care: How to make it real? A qualitative study among Dutch stakeholders BMC Fam Pract 2012 13 1 14 10.1186/1471-2296-13-14 22405260 PMC3355054

[B14] Moser A van der Bruggen H Widdershoven G Spreeuwenberg C Self-management of type 2 diabetes mellitus: a qualitative investigation from the perspective of participants in a nurse-led, shared-care programme in the Netherlands BMC Public Health 2008 8 1 91 10.1186/1471-2458-8-91 18366665 PMC2292711

[B15] Jonkers C Lamers F Bosma H Metsemakers J Kempen G Van Eijk J Process evaluation of a minimal psychological intervention to reduce depression in chronically ill elderly persons Patient Educ Couns 2007 68 3 252 257 10.1016/j.pec.2007.06.010 17686605

[B16] Jonkers CCM Lamers F Evers SMAA Bosma H Metsemakers JFM van Eijk JTM Economic evaluation of a minimal psychological intervention in chronically ill elderly patients with minor or mild to moderate depression: a randomised trial (the DELTA study) Int J Technol Assess Health Care 2009 25 4 497 504 10.1017/S026646230999050X 19845979

[B17] Lamers F Jonkers CCM Bosma H Kempen GIJM Meijer JAMJ Penninx BWJH Knottnerus JA van Eijk JTM A minimal psychological intervention in chronically ill elderly patients with depression: a randomised trial Psychother Psychosom 2010 79 4 217 226 10.1159/000313690 20424499

[B18] Lamers F Jonkers CCM Bosma H Knottnerus JA van Eijk JTM Treating depression in diabetes patients: does a minimal psychological intervention affect diabetes-specific quality of life and glycemic control? A randomized controlled trial J Adv Nur 2011 67 4 788 799 10.1111/j.1365-2648.2010.05540.x 21226754

[B19] Curran GM Bauer M Mittman B Pyne JM Stetler C Effectiveness-implementation hybrid designs: combining elements of clinical effectiveness and implementation research to enhance public health impact Med Care 2012 50 3 217 226 10.1097/MLR.0b013e3182408812 22310560 PMC3731143

[B20] Lamers F Jonkers CCM Bosma H Diederiks JPM van Eijk JTM Effectiveness and cost-effectiveness of a minimal psychological intervention to reduce non-severe depression in chronically ill elderly patients: the design of a randomised controlled trial BMC Public Health 2006 6 1 161 10.1186/1471-2458-6-161 16790039 PMC1555592

[B21] Durlak J DuPre E Implementation matters: a review of research on the influence of implementation on program outcomes and the factors affecting implementation Am J Community Psychol 2008 41 3 327 350 18322790 10.1007/s10464-008-9165-0

[B22] Braam C van Oostrom SH Terluin B Vasse R De Vet HCW Anema JR Validation study of a distress screener J Occup Rehabil 2009 19 3 231 237 10.1007/s10926-009-9178-z 19396529 PMC2712065

[B23] Terluin B van Marwijk HWJ Ader HJ de Vet HCW Penninx BWJH Hermens MLM van Boeijen CA van Balkom AJLM van der Klink JJL Stalman WAB The four-dimensional symptom questionnaire (4DSQ): a validation study of a multidimensional self-report questionnaire to assess distress, depression, anxiety and somatization BMC Psychiatry 2006 6 34 10.1186/1471-244X-6-34 16925825 PMC1590008

[B24] Van Eijk JTM Diederiks JPM Kempen GIJM Honig A Van der Meer K Brenninkmeijer WJM Development and feasibility of a nurse administered strategy on depression in community-dwelling patients with a chronic physical disease Patient Educ Couns 2004 54 1 87 94 10.1016/S0738-3991(03)00201-5 15210265

[B25] Mynors-Wallis L Problem solving treatment in general psychiatric practice Advan Psychiatr Treat 2001 7 417 425 10.1192/apt.7.6.417

[B26] De Hollander A Hoeymans N Melse J Van Oers J Polder J Zorg voor gezondheid - Volksgezondheid Toekomst Verkenning 2006 [Public Health Forecast 2006] 2006 Houten: Bohn Stafleu Van Loghum

[B27] De Jong A Van Duin C **Regionale prognose 2009–2040: vergrijzing en omslag in groei naar krimp [Local forecast 2009–2040: ageing and demographic change from growth to shrinkage]** Bevolkingstrends 2009 57 4 35 44

[B28] Penninx BW Beekman ATF Ormel J Kriegsman DM Boeke AJP Van Eijk JTM Deeg DJH Psychological Status among elderly people with chronic disease: does type of disease play a part? J Psychosom Res 1996 40 521 534 10.1016/0022-3999(95)00620-6 8803861

[B29] Tsiachristas A Hipple-Walters B Lemmens KMM Nieboer AP Rutten-van Mölken MPMH Towards integrated care for chronic conditions: Dutch policy developments to overcome the (financial) barriers Health Poli 2010 101 2 122 132 10.1016/j.healthpol.2010.10.013 21067841

[B30] Hulscher M Laurant M Grol R Grol R, Wensing M, Eccles M Process evaluation of change interventions Improving patient care the implementation of change in clinical practice 2005 Oxford: Elsevier Butterwork Heineman 256 272

[B31] Saunders RP Evans MH Joshi P Developing a process-evaluation plan for assessing health promotion program implementation: a How-to guide Health Promot Pract 2005 6 2 134 147 10.1177/1524839904273387 15855283

[B32] Fade S Using interpretative phenomenological analysis for public health nutrition and dietetic research: a practical guide Proc Nutr Soc 2004 63 4 647 653 10.1079/PNS2004398 15831138

[B33] Campbell MK Fayers PM Grimshaw JM Determinants of the intracluster correlation coefficient in cluster randomized trials: the case of implementation research Clin Trials 2005 2 2 99 107 10.1191/1740774505cn071oa 16279131

[B34] Tuinman MA Gazendam-Donofrio SM Hoekstra-Weebers JE Screening and referral for psychosocial distress in oncologic practice Cancer 2008 113 4 870 878 10.1002/cncr.23622 18618581

[B35] Polonsky WH Anderson BJ Lohrer PA Welch GW Jacobson AM Aponte JE Schwartz CE Assessment of diabetes-related distress Diabet Care 1995 18 754 760 10.2337/diacare.18.6.754 7555499

[B36] Snoek FJ Pouwer F Welch GW Polonsky WH Diabetes-related emotional distress in Dutch and U.S. diabetic patients: cross-cultural validity of the problem areas in diabetes scale Diabet Care 2000 23 9 1305 1309 10.2337/diacare.23.9.1305 10977023

[B37] Welch G Weinger K Anderson B Polonsky WH Responsiveness of the problem areas in diabetes (PAID) questionnaire Diabet Med 2003 20 1 69 72 10.1046/j.1464-5491.2003.00832.x 12519323

[B38] Ashworth M Robinson S Godfrey E Stepherd M Evans C Seed P Parmentier H Tylee A Measuring mental health outcomes in primary care: the psychometric properties of a new patient-generated outcome measure, ‘PSYCHLOPS’ (‘psychological outcome profiles’) Primary Care Mental Health 2005 3 4 261 270

[B39] Aaronson NK Muller M Cohen PD Essink-Bot ML Fekkes M Sanderman R Sprangers MA te Velde A Verrips E Translation, validation, and norming of the Dutch language version of the SF-36 Health Survey in community and chronic disease populations J Clin Epidemiol 1998 51 11 1055 1068 10.1016/S0895-4356(98)00097-3 9817123

[B40] Ware JE Kosinski M Keller S A 12-item short-form health survey: construction of scales and preliminary tests of reliability and validity Med Care 1996 34 3 220 233 10.1097/00005650-199603000-00003 8628042

[B41] Cardol M De Haan RJ De Jong BA van den Bos GA De Groot IJ Psychometric properties of the impact on participation and autonomy questionnaire Arch Phys Med Rehabil 2001 82 2 210 216 10.1053/apmr.2001.18218 11239312

[B42] ‘Impact on Participation and Autonomy’ (IPA): Manual to the English version IPA http://www.nivel.nl/pdf/INT-IPA-Manual.pdf

[B43] Hakkaart-van Roijen L Bouwmans C Manual Short Form-Health and Labour Questionnaire (SF-HLQ) 2007 Rotterdam, theNetherlands: Erasmus Universitair Medisch Centrum Rotterdam, Institute for Medical Technology Assessment

[B44] Petkov J Harvey P Battersby M The internal consistency and construct validity of the partners in health scale: validation of a patient rated chronic condition self-management measure Qual Life Res 2010 19 7 1079 1085 10.1007/s11136-010-9661-1 20437206

[B45] Bosscher RJ Smit JH Confirmatory factor analysis of the general self-efficacy scale Behav Res Ther 1998 36 3 339 343 10.1016/S0005-7967(98)00025-4 9642852

[B46] Sherer M Maddux JE Mercadante B Prentice-Dunn S Jacobs B Rogers RW **The self-efficacy scale: construction and validation.** Psychol Rep 1982 51 2 663 671 10.2466/pr0.1982.51.2.663

[B47] Cully JA Armento ME Mott J Nadorff MR Naik AD Stanley MA Sorocco KH Kunik ME Petersen NJ Kauth MR Brief cognitive behavioral therapy in primary care: a hybrid type 2 patient-randomized effectiveness implementation design Implement Sci 2012 7 1 64 10.1186/1748-5908-7-64 22784436 PMC3503767

